# Syndrome des antisynthétases compliqué d'une myocardite sévère

**DOI:** 10.11604/pamj.2015.21.4.6871

**Published:** 2015-05-04

**Authors:** Madiha Mahfoudhi, Amel Gaieb Battikh

**Affiliations:** 1 Service de Médecine Interne A, Hôpital Charles Nicolle, Tunis, Tunisie

**Keywords:** Dyspnée, syndrome des antisynthétases, myocardite, Dyspnea, antisynthetase syndrome, myocarditis

## Image en medicine

Le syndrome des antisynthétases correspond à l'association d'une myosite, une polyarthrite, un phénomène de Raynaud, une atteinte pulmonaire interstitielle et une hyperkératose fissurée des mains. Des anticorps de type «antisynthétases», en particulier l'anticorps anti-Jo1caractérise ce syndrome. L'atteinte cardiaque est rare, pouvant être parfois fatale. Patient âgé de 42 ans, hospitalisé pour des myalgies diffuses évoluant depuis un mois, une polyarthrite des genoux et des poignets, une dyspnée récente et un phénomène de Raynaud. L'examen physique a révélé une tachycardie sinusale à 110 battements /minute, un déficit musculaire prédominant aux ceintures, une hyperkératose fissurée des paumes des mains et des râles sous crépitants aux deux bases pulmonaires. L'examen biologique a montré un taux élevé des enzymes musculaires à 5 fois la normale, un syndrome inflammatoire, un bilan hépatique et rénal sans anomalies. L’électromyogramme a confirmé un tracé de type myogène. Le bilan immunologique a révélé la présence d'anticorps anti-Jo1. Les explorations fonctionnelles respiratoires ont conclut à un syndrome restrictif sévère. Le scanner thoracique a retrouvé un aspect en rayon de miel évocateur d'une fibrose pulmonaire. Un syndrome des antisynthétases a été évoqué. Le traitement s'est basé sur une corticothérapie associée aux boli de cyclophosphamide. L’évolution était marquée par l'amélioration des signes musculaires et articulaires. Devant la persistance de la tachycardie inexpliquée et l'aggravation de la dyspnée, des explorations cardiaques ont été réalisées montrant une myocardite compliquée d'une insuffisance cardiaque sévère. Le patient était rapidement décédé dans un tableau de défaillance cardiaque compliqué d'un état de choc.

**Figure 1:**
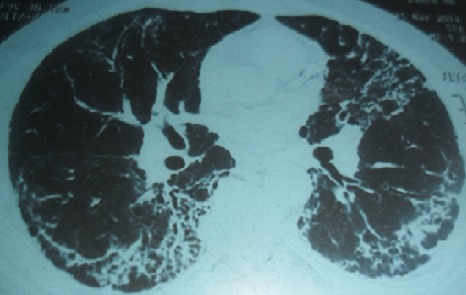
TDM thoracique: aspect en rayon de miel en faveur d'une fibrose pulmonaire

